# *Octopus vulgaris* Exhibits Interindividual Differences in Behavioural and Problem-Solving Performance

**DOI:** 10.3390/biology12121487

**Published:** 2023-12-04

**Authors:** Andrea Dissegna, Luciana Borrelli, Giovanna Ponte, Cinzia Chiandetti, Graziano Fiorito

**Affiliations:** 1 Department of Life Sciences, University of Trieste, 34127 Trieste, Italy; andrea.dissegna@units.it (A.D.); cchiandetti@units.it (C.C.); 2 Animal Physiology and Evolution Lab, Stazione Zoologica Anton Dohrn, Villa Comunale, 80121 Napoli, Italy; info@thesmartoctopus.com; 3 Department of Biology and Evolution of Marine Organisms, Stazione Zoologica Anton Dohrn, Villa Comunale, 80121 Napoli, Italy; giovanna.ponte@szn.it

**Keywords:** problem solving, extractive foraging, innovation, behavioural plasticity, seasonality, octopus, cephalopods, social learning, neophilia

## Abstract

**Simple Summary:**

Here, we investigated how *Octopus vulgaris* approached and solved a problem required for obtaining food from a puzzle box. We also explored the relationship between individual octopuses’ problem-solving abilities and various behavioural characteristics (e.g., their interest in novel objects), and biotic and environmental factors (age, season, and site of capture). We found that octopuses more inclined to approach new objects were quicker to approach the puzzle box and more likely to succeed in opening it, but they did not reach the solution before other individuals. This suggests that an excessive inclination towards novelty could hinder problem-solving efficiency. The study also revealed that the season and the fishing site are important drivers of octopuses’ behavioural differentiation. Our findings offer valuable insights into the individuality of octopuses.

**Abstract:**

By presenting individual *Octopus vulgaris* with an extractive foraging problem with a puzzle box, we examined the possible correlation between behavioural performances (e.g., ease of adaptation to captive conditions, prevalence of neophobic and neophilic behaviours, and propensity to learn individually or by observing conspecifics), biotic (body and brain size, age, sex) and abiotic (seasonality and place of origin) factors. We found more neophilic animals showing shorter latencies to approach the puzzle box and higher probability of solving the task; also, shorter times to solve the task were correlated with better performance on the individual learning task. However, the most neophilic octopuses that approached the puzzle box more quickly did not reach the solution earlier than other individuals, suggesting that strong neophilic tendency may lead to suboptimal performance at some stages of the problem-solving process. In addition, seasonal and environmental characteristics of location of origin appear to influence the rate of expression of individual traits central to problem solving. Overall, our analysis provides new insights into the traits associated with problem solving in invertebrates and highlights the presence of adaptive mechanisms that promote population-level changes in octopuses’ behavioural traits.

## 1. Introduction 

The striking behavioural abilities and flexibility of *Octopus vulgaris* and other cephalopods have populated literary and scientific domains for centuries [1,2,3,4,5,6,7,8,9,10,11]. The common octopus is one of the best studied animals among cephalopod molluscs, due to the physiology, richness of the behavioural repertoire, and marked learning capabilities [7,12,13,14,15,16,17,18,19,20,21,22]. 

The recent understanding of the complexity of its genome and related physiological adaptations, e.g., [23,24,25,26,27,28,29,30,31,32,33,34] further renewed interest in these animals and their biological plasticity. In addition, studies on the abilities and behavioural flexibility of cephalopods and the findings that these appear to be linked to fundamental behavioural traits—formerly investigated primarily in vertebrate animals—promoted a new era of studies about the cognitive abilities of these animals [7,9,11,35,36,37,38,39,40,41,42]. These traits include neophobia, problem solving, learning, and the social domain [43,44,45,46], to mention some. For example, a battery of experiments—carried out throughout standardized daily care protocols and behavioural paradigms—provided evidence that various individuals adopt distinct strategies to adapt to the captive condition, perform along a shy–bold continuum (neophobia), and solve problems and learn tasks with high interindividual heterogeneity [43,46]. Such individual differences that in octopuses are predominantly linked to predatory behaviour [14,15,47,48] may represent a shared trait across cephalopods’ cognitive processes, e.g., [7,37,49,50] and modes of life, e.g., [51,52,53,54,55,56]. 

### 1.1. Extractive Foraging Implies Sophisticated Problem-Solving Capacity

In many situations, food is hidden or not directly accessible to animals. To obtain resources for their survival, animals evolved various strategies including direct manipulation of the hiding place and/or tool use. In some cases, they specialized body parts, such as the arms of the octopus and other cephalopods, to facilitate the detection, capture, and manipulation of prey, with the constraint of not having a vertebrate mouth [17]. The organisms’ capacity to adapt to the external environment to reach inaccessible food is called extractive foraging, e.g., [57,58]. This ability plays a pivotal role in the survival of wild animals foraging in complex natural environments. Animals’ ability to solve problems can vary both between and within species. Extractive foraging represents the most common problem-solving situation occurring in nature, e.g., [59,60].

Several octopus species are known to use a hunting technique, the speculative pounce sensu Yarnall, [61], by means of which a target area (e.g., sand, rocks, or crevices) is covered and surrounded by the arms and interbrachial web expanded while typical changes in body patterns occur—i.e., the interbrachial web first blanches but then gradually darkens (review in [20]). This hunting technique is adopted in several cases while moving between different areas on the bottom surface (see descriptions and notes for *O. vulgaris* and *O. cyanea* in Borrelli and colleagues [20], and Yarnall [61]); speculative hunting is also reported for *Sepioteuthis sepioidea* [20]. Octopuses adopt speculative hunting to extract hidden food, for example, from bivalve shells [62] and generalize this hunting technique to laboratory situations where they are presented with a live crab in a jar, e.g., [63,64].

Extractive foraging has been extensively studied in relation to the cognitive abilities of animals; examples are known from birds [65,66,67], carnivores, e.g., [68,69], cetaceans, e.g., [70], different species of monkeys, e.g., [58,71,72], and of course, octopus [61,62,64]. More recently, the large differences in extractive foraging capacity among individuals within a given species have been investigated. 

Briefly, in the classic experimental paradigm, an individual is presented with a live prey enclosed in a box, an obstacle that an animal must overcome to reach the prey [65]. The task at hand is to perform the motor actions required to open the box, as well as the type and number of plugs to be opened (e.g., pull, screw, or shutter). Not all individuals are able to apply the full range of motor patterns required to open the box. 

In the case of *O. vulgaris*, about half of the individuals solve this problem on their first time [63]. However, the probability of success appears to be related to several circumstances such as individual experience and trial-and-error learning [63], a longer exposure to the testing environment [43,46], and social learning from other members of the same species [73,74]. Thus, octopuses who are not immediately able to solve an extractive foraging problem may eventually succeed in this task if other factors are considered. These factors act as facilitators (like social learning) or deterrents (i.e., neophobia) to determine the task success, e.g., [75,76,77]. 

Variations in neophilia and exploration behaviour are linked to an individual’s capacity to solve extractive foraging problems across a range of species [66,78,79,80,81,82,83,84,85]. The reasons for such variability in cognitive performance within species is unclear, but evidence suggests that interindividual differences in extractive foraging problems are related to the innovation success of a population. Indeed, during an extractive foraging problem, animals are assessed for their ability to invent new behaviours or change existing ones to solve a novel problem review in [86]; namely, they are assessed for their capacity to innovate. Cumulative evidence indicates that the predictors of performance in extractive foraging problems are comparable to those that influence the innovation rate in the wild [59]. This connection suggests that extractive foraging tasks can be an ecologically relevant experimental assay to test the mechanisms driving innovation and diffusion of novel foraging techniques [87,88].

### 1.2. Problem Solving Is Essential to Innovate a Predatory Strategy

Innovation—i.e., the ability to solve new problems or invent more efficient solutions to known problems—has driven the development of human civilization including technology; this ability is not unique to humans but is shared with birds and primates, e.g., [87,89]. After an innovation is discovered by an individual, it spreads among members of the population through social learning mechanisms, e.g., [90,91]. The social transmission of innovations has important ecological benefits for members of the population, who can exploit the new discovery to change inefficient behaviours [90]. 

Are all members of a population equally gifted innovators? We are all familiar with the fact that some people are better innovators than others. In humans, certain personality traits have been more commonly associated with people’s ability to innovate since childhood. For example, children who are open to new experiences are more likely to abandon a learnt strategy and invent new ways to solve a problem [92]. Similarly, individual-level characteristics distinguish good from bad innovators in other species. The key characteristics of successful innovators manifest in a wide range of behaviours, including an individual’s tendency to approach a new object, namely, its neophilia–neophobia, e.g., [93], its ability to observe and learn from other individuals, e.g., [94,95], and its persistence in the face of aversive behavioural outcomes [80,83].

From an evolutionary perspective, there is a functional link between individuals’ traits and their ability to innovate [90]. Innovations are the result of trade-offs, of balances between advantageous but conflicting cognitive and behavioural traits [96]. For example, successful innovators may be spurred to new behavioural solutions because they are more attracted to new situations. This attraction may lead innovators to be exposed to risky situations more often than neophobic individuals, e.g., [97,98]. The trade-off between innovation success and individual traits is determined by natural selection. Low predation pressure may favour the selection of proactive behaviours and promote innovation. When the predation risk increases, selection may favour neophobic behaviours over innovations to increase survival but see also [99]. Both these behavioural phenotypes must be maintained in the population to cope with these extremes [83,100,101,102,103,104].

The number of species to which this evolutionary explanation can apply is severely limited. This limitation is a consequence of the fact that most studies on this topic have been conducted on a restricted number of vertebrates. It is not known which behavioural traits predispose invertebrates to innovation. 

Here, we contribute to filling this gap by investigating the individual traits associated with innovation potential in the cephalopod mollusk *O. vulgaris*. Despite its molluscan nervous system, octopuses parallel the behaviour of vertebrate species in many situations that require complex skills such as individual and social learning [17,22,73,105], tool use [106], and problem solving [63,107]. Octopuses also have stable individual-level traits, e.g., [45,52], some of which appear related to developmental plasticity [53,54] like in vertebrates. Because the abilities of the octopus bear striking similarities to vertebrate cognitive abilities, it is an ideal model for studying innovation in invertebrates.

### 1.3. Aims of the Study

We capitalized on the ‘innovative problem-solving’ approach commonly employed in vertebrate studies to investigate the behavioural responses of octopuses to an extractive foraging task and evaluate their problem-solving abilities [59,108]. The problem usually requires animals to extract food from a puzzle box by manipulating the object with parts of their body or with the help of tools. This mimics a natural situation where food is inaccessible, for example, because it is embedded in a shell or a nut. Octopus can open a puzzle box by screwing or pulling a plug to reach a crab inside it, e.g., [46,63]. In order to measure individual-level behavioural traits of octopuses in relation to problem solving, we utilized a battery of experiments conducted under standardized conditions as originally described by Borrelli and coworkers [43,46]. 

Following a similar methodology adopted with vertebrates, we focused on a given number of “elements” considered informative of an individual’s general behaviour. These included the ease of adaptation to captive conditions, the prevalence of neophobic and neophilic behaviours, and the propensity to learn individually or through observations of conspecifics. We also considered a set of biotic and abiotic factors that are expected to influence the octopus’s behaviour in such situations. In particular, body size, estimated age, gender, season, and location of origin are all factors potentially exerting long-lasting effects on animals’ behaviour in general; for review, see for example [109]; and consequently influence their innovative problem-solving skills, e.g., [110,111]. Evidence that these factors correlate with the behaviour of *O. vulgaris* is still limited but see [43,46]. 

Finally, we modelled the latency of response and success rate of individual octopuses in the innovative problem-solving task using the biotic factors and behavioural characteristics as predictors.

## 2. Methods

This study is based on the analysis of data available to us, which was generated as part of a PhD project of Dr L. Borrelli [43]. Thus, this work meets the criteria for responsible use of animals in research, by using historical data/laboratory records and by the adoption of the 3Rs principle to cephalopods [112] as stated in the Directive 2010/63/EU. 

The behavioural and morphological data and the experiments on live octopuses were collected and carried out at least a decade prior to the entry into force of the Directive 2010/63/EU—which for the first time included cephalopods in the list of animals whose use is regulated for scientific purposes in EU Member States [113,114].

### 2.1. Subjects

*Octopus vulgaris* (N = 54; males = 32, females = 22) included in this work are those originally utilized for the PhD Thesis of Dr. L. Borrelli [43]. Octopuses were captured at various times during the years 2002 and 2004 from the wild in various locations of the Bay of Naples (Tyrrhenian Sea, Italy) by local fishermen adopting standardized methods of capture [115] (see also Supplementary Materials). The fishermen transported the live animals on the same morning to the laboratory at the ‘Octopus lab facility’ of the Stazione Zoologica Anton Dohrn (Italy). 

As described in Borrelli and coworkers [46], octopuses were sexed, weighed, and housed in individual tanks (60 × 100 × 50 cm) under natural conditions similar to those that occur at 3–4 m depth at sea. A transparent glass partition was placed between adjacent tanks to allow visual interaction during the social interaction paradigms. Illumination was provided according to the seasonal and daily circadian rhythm; for details, see [43,46]. All the experiments were videorecorded with a camera positioned in front of the tank. 

At the end of the experiment, animals were sacrificed to measure the brain size (weight of the total brain, including the supra- and sub-oesophageal, and left and right optic lobes) and collect other morphological information [43]; animal age was estimated by counting the number of growth rings in the octopus’s upper beak [116].

### 2.2. Experimental Plan

**Figure 1:**
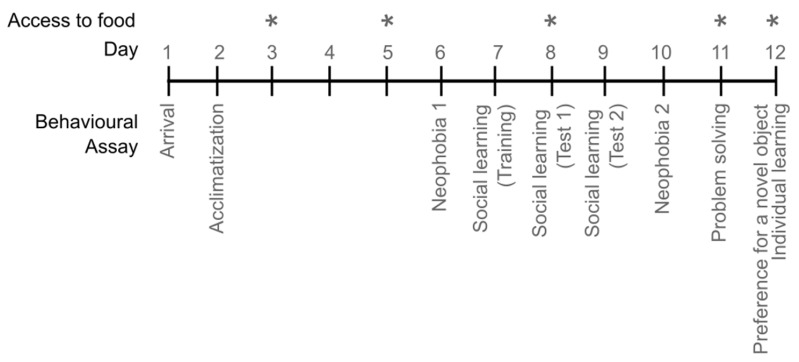
Schematic representation of the experimental plan and behavioural assays utilized in this study; asterisks indicate the feeding days (Adapted from Borrelli et al. 2020 [46]).

Octopuses were tested for 12 consecutive days from the day after their arrival (Day 1) in the laboratory, as described in Borrelli and colleagues [46]. The array of behavioural paradigms included in the study is listed below (see also Figure 1); for details, refer to Borrelli et al. [46] and the PhD Thesis by L. Borrelli [43] and Supplementary Materials.

(i) Acclimatization (Day 2 to 6)—Each animal was presented with a live crab (*Carcinus maenas*) and latency to attack was measured in seconds [14,48] for five consecutive days. Following Maldonado [14], this allows measurement of the recovery in an octopus’s predatory performance after its introduction to a controlled (i.e., captive) environment, and its natural attitude in response to a potential prey [48]. Octopuses were allowed to prey on the crab only on Day 2 and Day 5 according to the feeding regime established for this experiment (see the Supplemental Information); on all other occasions, the crab was removed by the experimenter for assessing the readiness to attack of *O. vulgaris* [14,47,48]. 

(ii) Neophobia (Days 6 and 10)—Following Greenberg [117,118], the neophobia tests were designed to compare the time required by an octopus to attack a crab when presented alone or with a novel object [43], i.e., providing a live crab alone (CRAB) or a live crab together with a novel object (CRAB&OBJ). Neophobia in *O. vulgaris* was assessed in two different circumstances: before (Neophobia 1st; Day 6) and after the Social Learning experiment (Neophobia 2nd; Day 10—for details, see Borrelli and coworkers [46]). A metallic cross (14 cm wide, 3 cm thick) was utilized in Neophobia 1st test; a metallic lid was used as a novel object during Neophobia 2nd. The difference in the latency (in seconds) to approach the prey/object when paired with the novel object by octopus was scored according to the formula: (Latency CRAB&OBJ—Latency CRAB)/(Latency CRAB&OBJ + Latency CRAB). 

(iii) Social Learning (Days 7 to 9)—On Day 7, the opaque partition that separated adjacent tanks was removed and octopuses (the tested individual, and a trained octopus; see [43]) could see each other through a transparent glass. On the following day, each octopus (observer) watched the trained conspecific (demonstrator) solving the problem of opening a black box to catch a crab hidden inside. Each observer octopus was then presented with the same box in a following trial (in isolation). Latency to approach, touch, open the box, and prey on the crab were scored. The same test was repeated the next day to investigate whether observers could benefit from repeated social experience with their demonstrators. For details, see Borrelli and coworkers [46].

(iv) Problem solving (Day 11)—As detailed in Borrelli et al. [46], octopuses were presented with a box (20 × 15 × 15 cm) made of clear Plexiglas and containing a live crab. The box had three plugs—one for each side—that provided access to the inside. Each plug could be opened in a different way: by pulling, screwing, and sliding. Following Fiorito and colleagues [63], we analysed the performance of *O. vulgaris* in the problem-solving task by identifying the main phases of behaviours exhibited by the octopuses. In particular, we focused on approach (latency to approach the box), attack (latency to first contact), the opening of the box (latency to open the box), and seizure of the crab (latency to prey).

(v) Preference for a novel object (Day 12)—Two balls of a different colour (red and white, 4 cm of diameter) were simultaneously presented to each octopus for five (5) trials. A food reward was attached to each ball. At each trial, octopuses obtained a reward from only one of the two balls, as the other was promptly removed by the experimenter. In this case, we scored latency to attack the ball (see [46] for details). 

(vi) Individual Learning (Day 12)—Octopuses were presented with the previously preferred ball (i.e., the one they had chosen more times during the Preference test), but this time at each occasion the stimulus was paired to an aversive stimulus (i.e., a mild electric shock [46]). We scored the latency to approach the ball over nine (9) consecutive trials.

### 2.3. Data Analysis

Raw data were centered (x_i_ − $\bar{x}$) to express individual performance in terms of deviations from the group average. A principal component analysis (PCA) was conducted on the Pearson’s correlation matrix between the 28 variables resulting from the Acclimatization, Neophobia, Social Learning, Preference for a novel object, and Individual Learning tasks (for the detailed list, see the Supplementary Materials). We determined the number of components to retain in the PCA using a parallel analysis. The resulting components were rotated according to an *oblimin* procedure. 

We evaluated the behaviour and performance of individual octopuses by analysing the factor scores obtained from PCA. These scores reflect the contribution of each component to the individual performance on each task. To assign factor scores to each octopus, we used the regression method.

We analysed the relationship between seasonality and site of origin (fishing site), factor scores, and morphometric factors (i.e., brain and body size) by means of robust correlation coefficients and *t*-test on maximum-likelihood estimator differences. These robust methods can accommodate outliers by reducing their influence in the statistical analysis without excluding individuals with extreme performance. Robust 95% confidence intervals of each effect size (Cohen’s *d*) were reported to facilitate the interpretation of the results.

Furthermore, we tested whether individual differences in factor scores and morphometric factors predicted octopuses’ ability in the problem-solving task. We used linear regression models based on maximum likelihood to model the latency distributions for approaching the box, attacking, opening of the box, and seizing the crab, and generalized linear models (with “logit” link) to predict the likelihood of success on this task. 

Finally, we performed a cluster analysis across factor scores (Acclimatization, Neophobia, Neophilia, and Social learning), morphometric factors (brain size), age, and problem-solving measures to identify groups of individuals with similar abiotic and biotic characteristics. We then performed a logistic regression to estimate the probability of an individual belonging to a particular cluster using the same predictors. To determine the most informative predictors influencing cluster membership, we applied a stepwise backward procedure, iteratively removing predictors that did not improve the model fit as determined by AIC. 

The analyses were performed using R [119]. PCA and parallel analysis were carried out using the *psych*() and the *paran*() packages, respectively; robust two-sample tests and correlations using the *WRS2*() package [120]; and robust regressions using the *MASS*() package [121]. The logistic regressions were performed using the *glm*() function and the stepwise backward procedure, with the function *step*() of the package *stats*(). Cluster analysis was performed using the *mclust*() package [122].

## 3. Results

Based on the outcomes of the data analysis, four factors were retained explaining 58% of the variance. The variables were grouped according to the specific ‘tasks’ included in the behavioural array of tests, indicating that the four Components were represented, ‘Component 1′: Social Learning; ‘Component 2′: Individual Learning; ‘Component 3′: Acclimatization; ‘Component 4′: Neophilia (for additional information, see Supplementary Materials). 

We found a significant correlation between Social Learning and Neophilia (*r* = 0.28, *p* = 0.022).

### 3.1. Morphometric and Behavioural Characteristics Associated with Seasonality

**Figure 2:**
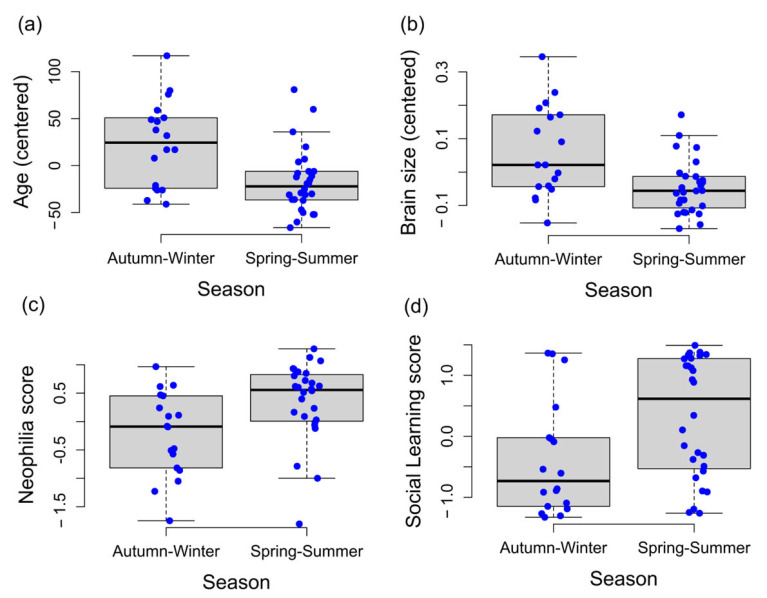
Boxplots for the estimated age (**a**), brain size (**b**), neophilia (**c**), and social learning scores (**d**) for *O. vulgaris* captured in autumn–winter or spring–summer. Blue dots represent individuals’ scores.

As shown in Figure 2, octopuses collected during spring–summer were overall younger (*t*(15.36) = 3.05, *p* = 0.007; *d* = 0.76, CI[0.26–1.98]) and had a smaller brain size (*t*(14) = 2.76, *p* = 0.015; *d* = 0.67, CI[0.19–1.22]) than *O. vulgaris* captured during autumn–winter seasons. Animals belonging to spring–summer seasons scored the highest in Neophilia (*t*(27.36) = 2.78, *p* = 0.012; *d* = 0.74, CI[0.18–1.51]) and Social Learning (*t*(27.36) = 2.88, *p* = 0.007; *d* = 0.97, CI[0.20–2.64]).

### 3.2. Morphometric and Behavioural Characteristics Associated with the Location of Origin (Fishing Site)

**Figure 3:**
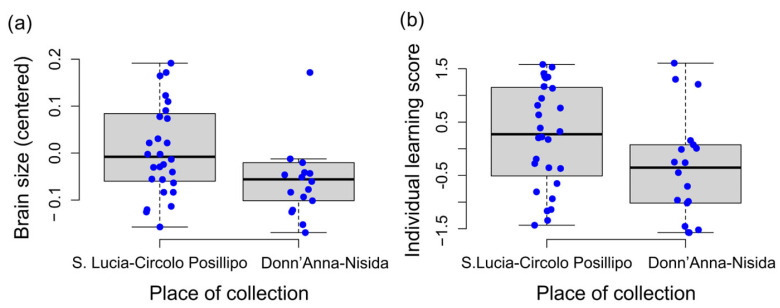
Boxplots of the brain size (**a**) and the Individual Learning score (**b**) of *O. vulgaris* captured in the S. Lucia-Circolo Posillipo or the Donn’Anna-Nisida areas in the Bay of Naples (Mediterranean Sea, Italy; see Supplementary Materials for details about the sites of capture). Blue dots represent individuals’ scores.

Octopuses collected in the S. Lucia–Circolo Posillipo area (Bay of Naples, Mediterranean Sea, Italy) had a larger brain size than those fished in the Donn’Anna-Nisida area (*t*(27.36) = 2.34, *p* = 0.027; *d* = 0.61, CI[0.03–1.22]; see Figure 3a). The age of the octopuses gathered at the two sites did not differ significantly (*t*(27.91) = 0.38, *p* = 0.707; *d* = 0.55, CI[−0.14–1.03]). In addition, *O. vulgaris* captured in the S. Lucia–Circolo Posillipo area performed with behaviours that scored high in Individual Learning, indicating fast avoidance learning (*t*(27.36) = 2.39, *p* = 0.023; *d* = 0.55, CI[0.30–0.99]; see Figure 3b).

### 3.3. Correlations between Morphometric and Individual Behavioural Differences

We found that the individual Neophilia score was negatively associated with animals’ age and brain size. Octopuses with high Neophilia scores were younger (*r* = −0.28, *p* = 0.047) and had a smaller brain (*r* = −0.35, *p* = 0.010). Among the morphometric variables considered, the brain size was positively correlated with the size of the supra-oesophageal mass (*r* = 0.68, *p* < 0.001), the body size (*r* = 0.31, *p =* 0.031), and the estimated age (*r* = 0.64, *p* < 0.001). 

### 3.4. Morphometric and Behavioural Characteristics Associated with Problem-Solving Skills

We ran a set of regression models to determine whether individual characteristics and morphometric features of octopuses explained their problem-solving performance. Separate models were run for each phase of the behavioural sequence leading to the solution of the problem, as described by Fiorito and colleagues [63]. 

Latency to approach the box resulted as being negatively associated with Neophilia score. In particular, the Latency to approach the box decreased from low to high Neophilia scores at a rate of *b* = −2.87 s (*se* = 1.41, *F* = 4.36, *p* = 0.043). This reveals that more neophilic *O. vulgaris* approached the box more quickly than less neophilic animals. Octopuses with a larger brain had shorter latency to make first contact with the box. The latency to first contact indeed increased from octopuses with a small brain to octopuses with a large brain at a rate of *b* = 24.09 s (*se* = 6.50, *F* = 12.46, *p* = 0.001). The latency to open the box was negatively associated with Individual Learning, as it decreased from low to high Individual Learning scores at a rate of *b* = −18.42 s (*se* = 7.81, *F* = 5.72, *p* = 0.021). Thus, octopuses who were better learners solved the task more quickly. 

Finally, the latency to seize the crab hidden in the box resulted to be negatively associated with Neophilia. In fact, the latency to seize the crab decreased from low to high Neophilia scores at a rate of *b* = −22.12 s (*se* = 8.83, *F* = 6.57, *p* = 0.015), revealing that more neophilic octopuses reached the prey more quickly.

Overall, the likelihood of success in the problem-solving task was positively associated with the Neophilia score (*b* = 1.21, *se* = 0.61, *z* = 1.97, *p* = 0.048), and negatively associated with the number of attempts to open the box by octopuses (*b* = −0.18, *se* = 0.07, *z* = −2.38, *p* = 0.017).

### 3.5. Grouping Individuals among Features

**Figure 4:**
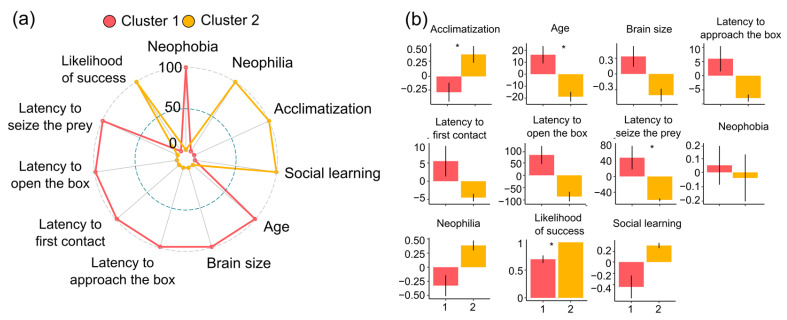
Spider plots (**a**) representing the score (normalized within the 0–100 range) of each variable in the two clusters. Barplots (**b**) of the corresponding means and standard error of the means (see the Supplementary Materials for the descriptive statistics of the two clusters). Asterisks indicate significant differences between clusters (*p* < 0.050).

Figure 4 summarizes the results of the cluster analysis (for details, see also Table S2 in Supplementary Materials). Examination of Bayesian information criterion (BIC) indicated that a two-cluster solution was the best-fitting model (BIC = −2984.89). Both clusters were composed of an equal number of individuals (*n* = 23), but they grouped octopuses with opposite characteristics. Individuals in Cluster 1 were characterized by higher neophobia and lower neophilia, thus confirming that the tasks employed were sensitive in measuring reciprocal behavioural traits. Octopuses took longer to adjust to a new environment and had poorer social learning skills; on average, they were older and had larger brains. Individuals in this cluster tended to perform poorly in all stages of problem-solving and had lower success rates. In contrast, individuals grouped in Cluster 2 were less neophobic and more neophilic. They adapted quickly to new environments and were better social learners, on average. These octopuses were younger and had smaller brains, and were generally faster in completing all phases of the problem-solving task, and successful in finding the solution.

We used a logistic regression with a stepwise backward procedure to identify the most informative predictors of cluster membership (AIC = 24). The resulting model included acclimatization, age, brain size, latency to seize the crab, and likelihood of success in the problem-solving task. Acclimatization (*b* = 2.29, *se* = 0.95, *p* = 0.015) and the likelihood of success in the task (*b* = 9.26, *se* = 3.38, *p* = 0.006) were positively associated with membership in Cluster 2, consistent with the observation that individuals in this cluster acclimatized earlier and were more successful at the task. Age (*b* = −0.10, *se* = 0.04, *p* = 0.011) and latency to seize the crab (*b* = −0.02, *se* = 0.01, *p* = 0.009) were negatively associated with this cluster, in line with the observation that individuals in this cluster were younger and faster in seizing the crab. 

These findings suggest that these four factors were more influential in determining cluster membership than other ones. In the two clusters, the relative frequency of grouping of individual octopuses did not differ for gender, fishing sites, and seasons (all *p* > 0.09).

## 4. Discussion

Here we described the individual-level characteristics that correlate with octopuses’ performance on the extractive foraging problem. The same traits characterize successful innovators also in vertebrates, thus suggesting a continuum in the animal kingdom between the individual-level traits associated with individual innovation success and supporting an evolutionary explanation for individual differences in innovation, e.g., [46,59,63,66,123,124,125,126,127,128].

In *O. vulgaris*, innovative problem solving is not a unitary phenomenon; rather, it involves multiple cognitive tasks that must be performed to achieve the overall solution of the problem. This is corroborated by the fact that the distinct individual-level characteristics of octopuses influence distinct stages of the problem-solving process. 

Neophilia was positively correlated with the octopuses’ latency to approach the puzzle box and the probability of reaching the solution of the task, while individual learning was positively correlated with the time to solve the task. A key finding of our experiment is that strong neophilic tendencies can lead to suboptimal performance at some phases of the problem-solving process. Neophilic octopuses approached the puzzle box more quickly, but despite this initial advantage, they did not reach the solution earlier than other individuals. This could be due to the fact that once an octopus has approached the box, it needs to collect relevant cues to open the plugs. At this stage of the process, individual learning ability is more important, as shown by the fact that octopuses with greater learning ability had an ‘advantage’. Our data suggest that there is a trade-off between speed and accuracy in the resolution of the problem-solving task. Some animals may trade off the speed of approach to a new object for better sampling and collecting information relevant to solving the task [82,129,130], suggesting less efficient information processing in more proactive individuals, e.g., [91]. 

Overall, the comparison of octopuses’ performances across tasks and other factors considered resulted in two clusters of individuals with opposite characteristics (Figure 3). In particular, the composition of the two clusters seems to reflect a superordinate distinction between reactive and proactive individuals, with Cluster 1 representing more reactive individuals and Cluster 2 representing more proactive octopuses. Reactive individuals were characterized by higher neophobia, lower neophilia, longer adaptation time to captive conditions, and poorer social learning skills, while proactive individuals exhibited the opposite characteristics. Our results hint at the possibility of classifying individuals along a continuum (reactive to proactive), and provide a framework for understanding the diversity of behavioural strategies in octopus and cephalopods in general. By studying differences within this continuum, it is also possible to gain insights into how these animals respond to environmental challenges and opportunities. For example, such a behavioural characterization may improve understanding and management of octopus welfare in captivity. Proactive individuals may result to be more flexible (adaptable) to new environments and less prone to stressful events, while reactive individuals may require more careful management to ensure their well-being. By profiling individual octopuses, researchers and caretakers could tailor their management strategies to better meet their individual needs. 

Additionally, we found a combination of biotic factors, such as acclimatization, age, latency to preying on the crab, and likelihood of success in the problem-solving task, that were responsible for clustering reactive and proactive octopuses. These factors may be important targets for future research on personality in cephalopods.

At the population level, the seasonality and the environmental main characteristics of the site of origin correlate with the expression rate of octopus traits. Animals captured in spring and summer were more neophilic and better “social” learners than octopuses collected in autumn and winter. Moreover, octopuses collected in the area from Santa Lucia to Circolo Posillipo in the Bay of Naples (Mediterranean Sea) had larger brains and learned better in non-social tasks than octopuses collected from Donn’Anna to Nisida areas in the Bay of Naples. These results indicate that there are adaptive mechanisms that modulate the morphological and behavioural characteristics of the octopus population to favour niche specialization, as was recently shown in other animals [83,131,132,133]. 

We can only speculate about the nature of the mechanisms involved. Several studies have shown that juveniles are often more explorative and neophilic than adults [134,135,136,137,138,139,140]. Hence, the seasonal difference in neophilia and social learning abilities may be related to the prevalence of younger octopuses during spring–summer. During this period, the autotrophic biomass in the Gulf of Naples peaks and resources become abundant [141,142]. Octopuses may also benefit from the increased willingness to approach new objects and take more risks during spring–summer to collect more resources [46]. In addition, the environmental characteristics of the two sites (see Supplementary Materials) revealed that the area from Santa Lucia to Circolo Posillipo has a much more uniform seabed than the area from Donn’Anna to Nisida. The seabed from Donn’Anna to Nisida may present an enriched environment that can enhance octopus learning.

## 5. Conclusions

Successful innovators may be characterized by certain behavioural characteristics that have been reported previously in vertebrates. Here, we have documented these in individuals of *O. vulgaris* that were able to solve the innovative problem-solving task. This provides important hints to the evolutionary history of the characteristics that are more likely to be associated with problem solving and innovation. Moreover, we documented differences among ‘reactive’ and ‘proactive’ octopuses, which may have implications for octopuses’ welfare and management in captivity. We also identified key characteristics that distinguish reactive from proactive individuals and may be important targets for future research on octopus personality. Finally, we have shown that seasonal and geomorphological factors of the location of origin of octopus alter the expression rate of individual traits that are central to problem solving. 

This suggests the presence of adaptive mechanisms that promote changes in octopuses’ behavioural traits at the population level.

## Data Availability

All data included in this study are available in the main text and Supplementary Information. Raw data are available from the Open Science Framework Repository: https://osf.io/dqkfm/ (accessed on 2 December 2023).

## Supplementary Materials

The supporting information can be downloaded at: https://www.mdpi.com/article/10.3390/biology12121487/s1. These include details for sites of capture of *O. vulgaris* [143,144,145,146], and an outline of the experimental procedures. Figure S1: Sites of capture. The Gulf of Naples in an old hand drawing by S. Ranzi [147] and an outline of the area of interest where octopuses have been captured. Ranzi’s drawing includes main isobaths. Modified from Ranzi, 1930 [147]. Outline of the Bay of Naples (Bottom) with the location (pink shaded areas) of the fishing sites where octopuses were caught [43].; Tables S1–S3: pattern matrix resulting from PCA with standardized loadings based upon correlation matrix after PCA (TC1 to TC4: Social Learning, Individual Learning, Acclimatization, and Neophilia, respectively), and Variance and Correlation matrices resulting from the PCA. Cluster analysis: descriptive statistics of the two clusters resulted from the analysis (Table S4) [148,149].
