# Supplementary material for: Octopus vulgaris Exhibits Interindividual Differences in Behavioural and Problem-Solving Performance
Source: Biology (Basel). 2023 Dec 4;12(12):1487. doi: 10.3390/biology12121487 (PMC10740590; doi:10.3390/biology12121487)
Supplement: Supplementary file 1 [file biology-12-01487-s001.zip › biology-2716437-supplementary.pdf]

Supplementary Info to

Octopus vulgaris Exhibits Interindividual Differences in Behavioural and Problem-Solving Performance

Andrea Dissegna 1, Luciana Borrelli 2, Giovanna Ponte 3, Cinzia Chiandetti 1 and Graziano Fiorito 3,*

1 Department of Life Sciences, University of Trieste, 34127 Trieste, Italy; andrea.dissegna@units.it (A.D.); cchi-andetti@units.it (C.C.)

2 Animal Physiology and Evolution Lab, Stazione Zoologica Anton Dohrn, Villa Comunale, 80121 Napoli, Italy; info@thesmartoctopus.com

3 Department of Biology and Evolution of Marine Organisms, Stazione Zoologica Anton Dohrn, Villa Comunale, 80121 Napoli, Italy; giovanna.ponte@szn.it

* Correspondence: graziano.fiorito@szn.it

Table of Contents

Sites of capture 3

Outline of the experimental procedure 5

Principal Component Analysis 8

Supplementary Table 1 – Pattern Matrix resulting from PCA 8

Supplementary Table 2 - Variance matrix 9

Supplementary Table 3 – Correlation matrix 9

Cluster Analysis 10

Supplementary Table 4 **-** Descriptive statistics 10

References 11

Sites of capture

Octopus vulgaris tested in the experiments included in this study originate from the PhD project of Dr L. Borrelli [1]. Animals were collected from the Bay of Naples (Mediterranean Sea, Italy) during years 2002 and 2003, at a depth ranging from 2-30 m and at a distance from the coastline ranging from 0-900 m.

In order to standardize capture method a fisherman, Mr Antonio Di Liello, caught all animals. The octopuses were collected from various locations of the Bay of Naples (Thyrrenian Sea) i.e. from Nisida to S. Lucia (Supplementary Figure 1).

Proceeding along the coast, from West to East, one octopus was caught at Nisida, three at Marechiaro, ten at Palazzo Donn'Anna, twenty-two (22) octopuses at Circolo Posillipo, four at Mergellina, and 11 at S. Lucia. In a few cases (four animals) local fishermen captured the animals probably from Nisida. *O. vulgaris* included in the original sample were thus slightly biased among geographical sites since most of the animals were collected from Circolo Posillipo (40%), Donn'Anna (18%), and S. Lucia (20%), and only 22% came from other fishing sites.

Information on the geomorphology and on the floral and faunal assemblages of the coastal areas of the Gulf of Naples is outdated [2-4] lacking and studies are in progress to fill this gap [e.g., 5]. As a consequence, our present knowledge on the nature and richness of the environment where the octopuses were collected from is scarce and fragmented.

The original findings [i.e., the behavioural performance of octopuses in various tasks resulted to vary significantly among geographical sites of origin, see 1] were supported by a characterization of the faunal assemblage of the different fishing sites by L. Borrelli and A. Mercorella who reviewed the literature available in search of information relative to the diversity of prey-items of *O. vulgaris* among geographical sites in the Bay of Naples [1,5].[1]

In brief, and following Borrelli [1], the seascape and environment to the NW of the Bay of Naples appeared more diverse and with a richer faunal assemblage than the one surrounding Castel dell'Ovo (Suppl. Fig. 1).

[1] see Mercorella et al. (in press) Potential diversity of *Octopus vulgaris* living environments in the Bay of Naples (Italy) available in: *CephRes Reference Docs*

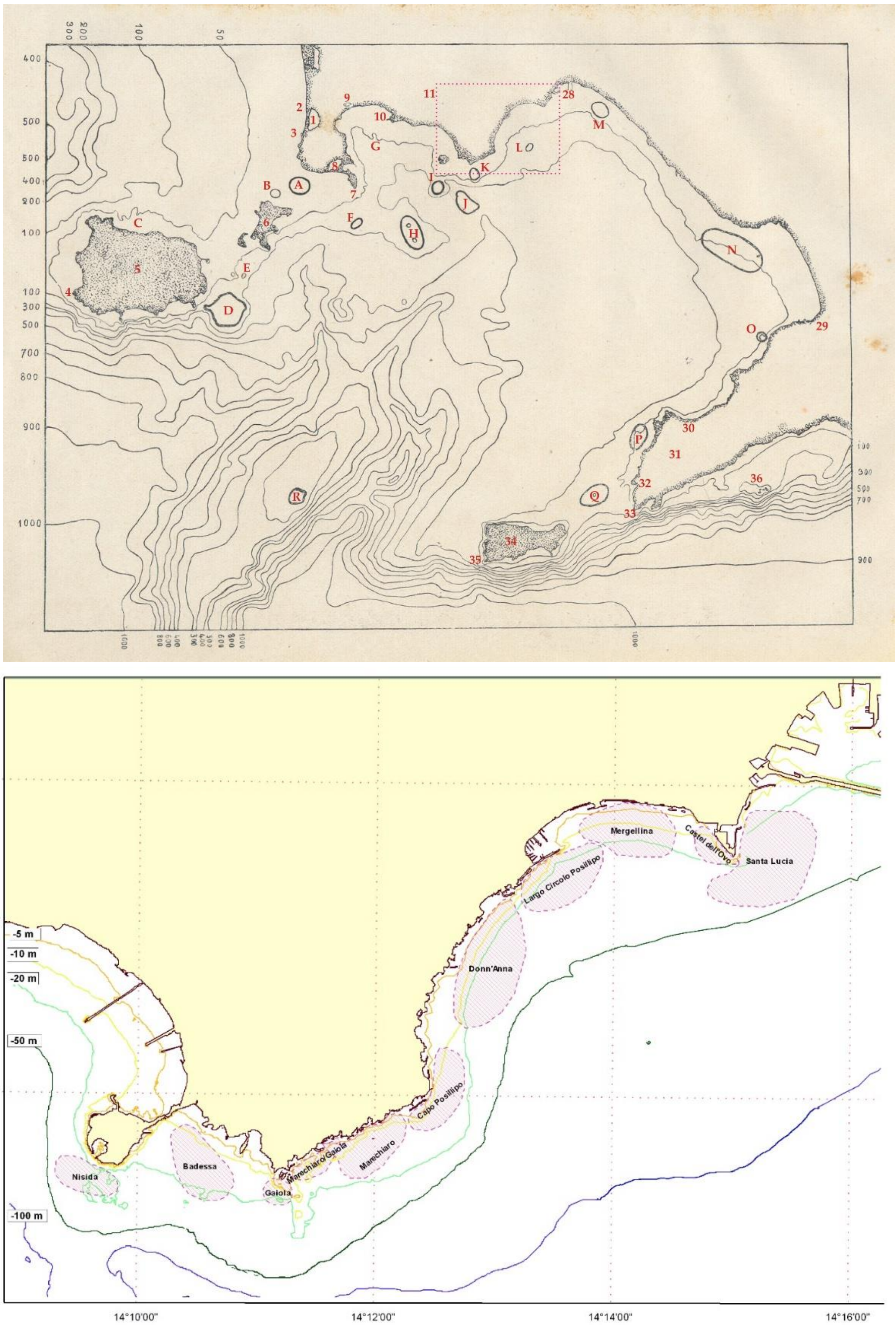

Figure S1. The Gulf of Naples in an old hand drawing by S. Ranzi (**Top**) and an outline of the area of interest (**Bottom**) where octopuses have been captured (magenta rectangle). Ranzi's drawing includes main isobaths. Main coastal areas (including islands, banks and shoals) are highlighted. **Coastal areas and islands**: *1.* Lake Fusaro; *2.* Fusaro beach; *3.* Torregaveta; *4.* Punta Imperatore; *5.* Island of Ischia; *6.* Island of Procida; *7.* Capo Miseno; *8.* Mar Morto; *9.* Lake Lucrino; *10.* Pozzuoli; *11.* Campi Flegrei; *12.* Nisida; *13.* Porto Paone; *14.* Badessa; *15.* Trentaremi; *16.* La Gaiola; *17.* Marechiaro; *18.* Capo Posillipo; *19.* Posillipo; *20.* Cenito; *21.* Donn'Anna; *22.* Mergellina; *28.* Naples harbour; *29.* Castellammare; *30.* Sorrento; *31.* Sorrentine Peninsula; *32.* Mitigliano bight; *33.* Punta della Campanella; *34.* Island of Capri; *35.* Punta Carena; *36.* li Galli. **Banks, shoals**: *A.* Torrione shoal; *B*. Marseglia shoal; *C*. Casamicciola bank; *D.* Ischia bank (also called Chiana bank); *E.* "La Catena" rocks; *F.* Capo Miseno bank (also called "Secca delle Prete nere"); *G.* Pozzuoli bank; *H.* Benda Palummo bank; *I.* Nisida shoal; *J.* Ammontatura trough; *K.* La Gaiola shoal; *L.* Deep rocks of Chiaia; *M.* Submerged artificial rocky reef of S. Giovanni a Teduccio; *N.* Torre Annunziata shoal; *O.* Vico Equense shoal; *P.* Vervece rock; *Q.* Bocca Piccola bank; *R.* Bocca Grande bank. Modified from Ranzi, 1930 [6]. Outline of the Bay of Naples (**Bottom**) with the location (pink shaded areas) of the fishing sites where octopuses were caught (Borrelli, 2007 [1]).

The sea bottom of the latter is mainly occupied by dead "matte" of *Posidonia oceanica*, which would seem to indicate that bottom cover was once more extensive and continuous. Moreover, in these dull surroundings, only a few bivalve species are abundant (G. Russo, pers. comm. to L. Borrelli [1]). On the basis of the information above (see also Mercorella and coworkers[1]), the animals were grouped in two broad areas of origin (Site 1 and Site 2) according to the similarities in the type of sea bottom (i.e. geomorphology and faunal diversity) of the two areas [P. Sansone, pers. comm. to G. Fiorito; 1]. A mixed sandy layer (i.e., coarse and fine-grained) has been reported to characterize the seabed, which extends from S. Lucia to Mergellina and Circolo Posillipo (identified as Site 1). This uniform seascape seemed to have a few exceptions: ***i.*** in the surroundings of Castel dell'Ovo where rubble gradually mixes offshore with coarse sand; ***ii.*** in Mergellina where fine sand is mixed to mud, and ***iii.*** at Circolo Posillipo where the fine-grained sand is substituted by pebbles. Finally, the deep rocks of Chiaia are the sole "shoal" of this area [2-4]. In contrast, the coastline that extends from Donn'Anna to Nisida (identified as Site 2) is characterized by a more diversified seabed with pebbles and scattered rocks, where shoals (La Gaiola shoal, Nisida shoal) and slopes (Ammontatura trough) alternate to areas of fine sand that are rich in Greek-Roman ruins (Marechiaro, La Gaiola-Trentaremi, Badessa).

Outline of the experimental procedure

On arrival in the laboratory, each *O. vulgaris* was identified (numbered), sexed, weighed, and housed in an experimental tank with running seawater [for details see: 1,7].

Following Fiorito and co-workers, the experimental setting was designed in order to simulate natural conditions at 3-4 m depth [8,9], despite the limitations of captive conditions [10,11]. In brief, tanks were located in a room, entirely painted dark turquoise (SIGMA coatings S-7020-B30G; colour components: Yellow = 30, Black = 50, Blue = 100), to which access was possible to experimenters only. The tanks (60 x 100 x 50 cm) made of dark grey PVC (colour components: Magenta = 10, Black = 50, Blue = 40) except for the front side that consists of a transparent glass panel (45 x 35 cm) to allow remote observation and videorecording. A yellowish-brown layer of sand (355-500 µm

granular size, 1 cm layer thickness), obtained directly from the coast off La Gaiola-Posillipo (i.e. Cenito; see #20 in Supplementary Fig. 1), was adjusted on the bottom of each tank and a pair of bricks, set in a corner, served as the octopus' den. All the tanks were firmly closed by a transparent cover (Plexiglas) with a hole to allow seawater inflow; the seawater is filtered before inflow to avoid sediment accumulation. At 1.40 m from the top of the tanks, three series of lamps (one series of Neodymlite tungsten ND60E27, two series of Neodymlite dichroic halogen MR16, Oy Airam AB, Finland) are positioned and programmed to switch on and off automatically according to the seasonal and daily rhythm at the latitude of the Bay of Naples. Each tank is paired with the adjacent one by a transparent glass partition, allowing visual interaction during social learning phases. In all other cases, each animal is kept in isolation by an opaque panel slid between the two tanks to cover the glass partition. A dark blue curtain (colour components: Magenta = 50, Black = 70, Blue = 100), dropping from the ceiling to the floor and running the entire length of the tanks, was positioned at a distance of 1.5 m from the frontal glass of each tank to hide both video-equipment (video-cameras, tripods, etc.) and the experimenter from the animals' view; the curtain has a series of slits through which only the lens of the video-camera is pulled through allowing video-recordings. On each tank, a second curtain drops from the ceiling to the surface of the water at the level of the frontal glass. These curtains have a similar brightness to the tank walls and help in hiding the tester during the experiments [details in: 1,7].

Each octopus was faced with a series of eight consecutive experiments presented to all the animals in the same order and lasting 12 days. The array of different paradigms always started on a Tuesday (Day 1) and ended the following Saturday (Day 12) and the experiments were arranged as follows: Arrival (Day 1), Acclimatization (Days 2-6), Neophobia 1st (Day 6), Social interaction (Day 7), Social learning (first session; Day 8), Social learning (second session; Day 9), Neophobia 2nd (Day 10), Innovation (Day 11), Preferences, Individual learning (Day 12).

Feeding regime was set in accordance with the standard maintenance procedures followed by Dr Fiorito's laboratory at the Stazione Zoologica, i.e. animals are fed every other day [1,7-9], with few exceptions: to avoid interference between consecutive experiments and set equal conditions between paradigms (e.g. Neophobia 1st and 2nd) animals were fed on Day 3, Day 5, Day 8, Day 11. Under these conditions, octopuses did not show neither physiological nor motivational decline to attack live prey [1,7-9]. Ceiling latencies were assigned for each experiment, either on the basis of

previous experience with the training paradigm or following the average performance of animals derived from a series of pilot studies designed ad hoc by Dr L. Borrelli (data not shown).

For Acclimatization a ceiling latency of 301 s was assigned to animals that failed to respond within a 5-minutes interval (trial duration), following which the crab was pulled out and the trial ended. On feeding days (i.e. Days 3, 5) animals that did not attack the crab within the ceiling latency were not fed. As a general criterion, the paradigms following Acclimatization were carried out only on animals that succeeded in recovering their predatory behaviour: i.e. by attacking a crab readily by the end of the five days. The other individual animals that did not meet this criterion are not included in this study [for details see 1].

Upon reception, each octopus was numbered, sexed, weighed (scale: Avery Berkel, mod. 342), and housed in an experimental tank (Fig. 1.1).

Principal Component Analysis

Table S1 – Pattern Matrix resulting from PCA

Standardized loadings (pattern matrix) based upon correlation matrix after PCA. TC1 to TC4 corresponds to Social Learning, Individual Learning, Acclimatization, and Neophilia, respectively.

h2: commonality, the amount of variance in the item/variable explained by the (retained) factors; i.e. the sum of the squared loadings. **u2**: uniqueness, the residual variance (u2 = 1 - h2). **com**: item complexity; Hoffman's index of complexity for each item. Other abbreviations: **Soc**: Social Learning; **IND**: Individual Learning; **Neo**: Neophobia; **PR**: Preference; **Accl**: Acclimatization; **LatOpen**: Latency to Open; **LatApprBox**: Latency to Approach the box; **LatAtt**: Latency to Attack the object (first contact); **LatPrey**: Latency to feed from the prey. See main text for details.

	item	TC1	TC2	TC4	TC3	h2	u2	com
Soc_S1_LatOpen_cent	7	0.93				0.871	0.13	1.1
Soc_S1_LatPrey_cent	9	0.93				0.875	0.13	1
Soc_S2_LatPrey_cent	13	0.91				0.856	0.14	1.1
Soc_S2_LatOpen_cent	12	0.91				0.857	0.14	1.1
Soc_S1_LatApprBox_cent	8	0.86				0.773	0.23	1.2
Soc_S1_LatAtt_cent	6	0.85				0.785	0.21	1.1
Soc_S2_LatAtt_cent	11	0.71				0.728	0.27	1.4
Soc_S2_LatApprBox_cent	10	0.63				0.668	0.33	1.6
IND_LatAtt6_cent	24		0.82			0.65	0.35	1
IND_LatAtt5_cent	23		0.76			0.67	0.33	1.2
IND_LatAtt3_cent	21		0.63			0.604	0.4	1.6
IND_LatAtt4_cent	22		0.62			0.594	0.41	1.7
Neo_incrementBaset1_cent	28		0.57			0.387	0.61	1.7
IND_LatAtt8_cent	26		0.56			0.364	0.64	1.2
IND_LatAtt7_cent	25		0.51			0.289	0.71	1.5
IND_LatAtt1_cent	19			0.78		0.669	0.33	1
PR_LatAtt4_cent	17			0.6		0.411	0.59	1.1
PR_LatAtt1_cent	14			0.55		0.632	0.37	2.3
PR_LatAtt5_cent	18			0.55		0.277	0.72	1.2
IND_LatAtt2_cent	20		0.47	0.52		0.659	0.34	2.2
PR_LatAtt3_cent	16			0.52		0.265	0.73	1
PR_LatAtt2_cent	15			0.44		0.456	0.54	2.3
Accl_LatAtt2_cent	2				0.91	0.86	0.14	1
Accl_LatAtt4_cent	4				0.81	0.726	0.27	1.1
Accl_LatAtt1_cent	1				0.72	0.551	0.45	1.5
Accl_LatAtt3_cent	3				0.65	0.535	0.47	1.7
IND_LatAtt9_cent	27					0.103	0.9	1.5
Accl_LatAtt5_cent	5					0.056	0.94	1.6

Table S2 - Variance matrix

The variance matrix resulting from the PCA. See main text for details.

	TC1	TC2	TC4	TC3
SS loadings	6.29	3.6	3.32	2.96
Proportion Var	0.22	0.13	0.12	0.11
Cumulative Var	0.22	0.35	0.47	0.58
Proportion Explained	0.39	0.22	0.21	0.18
Cumulative Proportion	0.39	0.61	0.82	1

Table S3 – Correlation matrix

The correlation matrix of the four components resulting from the PCA. See main text for details.

	TC1	TC2	TC4	TC3
TC1	1	0.21	0.27	0.16
TC2	0.21	1	0.19	0.04
TC4	0.27	0.19	1	-0.04
TC3	0.16	0.04	-0.04	1

Cluster Analysis

Table S4 - Descriptive statistics

Descriptive statistics of the two clusters resulted from the analysis. See main text for details. Means and standard errors of the means of *O. vulgaris* scores along each variable are reported.

Cluster	Acclimatization	Age	Brain size	Latency to Approach the box	Latency to first contact	Latency to open the box	Latency to seize the crab	Neophobia	Neophilia	Likelihood of success	Social learning
Cluster 1	-0.25±0.15	16.3±7.27	0.03±0.02	6.02±4.48	5.51±4.19	82.4±36.6	47.3±29.8	0.05±0.13	-0.33±0.18	0.69±0.07	-0.44±0.20
Cluster 2	0.36±0.14	-18.8±4.09	-0.04±0.01	-7.91±1.25	-4.51±1.09	-86.1±19.7	-59.4±3.83	-0.04±0.16	0.38±0.08	1	0.29±0.04

References

1. Borrelli, L. Testing the contribution of relative brain size and learning capabilities on the evolution of *Octopus vulgaris* and other cephalopods. PhD Thesis, Stazione Zoologica Anton Dohrn, Italy & Open University, UK, **2007**.

2. Colombo, A. La fauna sottomarina del Golfo di Napoli. *Rivista Marittima* **1888**, *Ottobre-Dicembre*, 5-107.

3. Parenzan, P. Ricerche sulle biocenosi del Golfo di Napoli. *Atti Società Italiana per il Progresso delle Scienze* **1933**, *III*, 1-3.

4. Parenzan, P. Contributo alla conoscenza delle elevazioni sottomarine del Golfo di Napoli. *Bollettino della Società dei Naturalisti in Napoli* **1954**, *LXIII*, 45-72.

5. Gambi, M.C.; D'Ambra, I.; Fiorito, G.; Saggiomo, V. The Archivio Moncharmont: a pioneering biodiversity assessment in the Gulf of Naples (Italy). *Oceanography in the Mediterranean and Beyond Pubblicazioni della Stazione Zoologica di Napoli* **2013**, *4*, 459-467.

6. Ranzi, S. La distribuzione della vita nel Golfo di Napoli. *Atti del XI Congresso Geografico Italiano* **1930**, *II*, 1-4.

7. Borrelli, L.; Chiandetti, C.; Fiorito, G. A standardized battery of tests to measure *Octopus vulgaris*' behavioural performance. *Invertebrate Neuroscience* **2020**, *20*, 4, doi:10.1007/s10158-020-0237-7.

8. Fiorito, G.; von Planta, C.; Scotto, P. Problem solving ability of *Octopus vulgaris* Lamarck (Mollusca, Cephalopoda). *Behavioral and Neural Biology* **1990**, *53*, 217-230.

9. Fiorito, G.; Biederman, G.B.; Davey, V.A.; Gherardi, F. The role of stimulus preexposure in problem solving by *Octopus vulgaris*. *Anim Cogn* **1998**, *1*, 107-112.

10. Grimpe, G. Pflege, Behandlung und Zucht der Cephalopoden fur zoologische und physiologische Zwecke. In *Handbuch der biologischen Arbeitsmethoden*, Äberhalden, E., Ed. Verlag Urban & Schwarzenberg: Berlin, Wien, **1928**; pp. 331-402.

11. De Sio, F.; Hanke, F.D.; Warnke, K.; Marazia, C.; Galligioni, V.; Fiorito, G.; Stravidou, I.; Ponte, G. E Pluribus Octo – Building Consensus on Standards of Care and Experimentation in Cephalopod Research; a Historical Outlook. *Frontiers in Physiology* **2020**, *11*, doi:10.3389/fphys.2020.00645.
